# Sociodemographic predictors of patients with brain metastases treated with stereotactic radiosurgery

**DOI:** 10.18632/oncotarget.22291

**Published:** 2017-11-07

**Authors:** Natalie Alphonse-Sullivan, Glen B. Taksler, Thomas Lycan, Kathryn E. Weaver, Emory R. McTyre, Rachel F. Shenker, Brandi R. Page, Scott Isom, Adam Johnson, Michael T. Munley, Adrian W. Laxton, Stephen B. Tatter, Kounosuke Watabe, Michael D. Chan, Jimmy Ruiz

**Affiliations:** ^1^ Department of Radiation Oncology, Wake Forest School of Medicine, Winston-Salem, NC, USA; ^2^ Medicine Institute, Cleveland Clinic Foundation, Cleveland, OH, USA; ^3^ Department of Medicine (Hematology and Oncology), Wake Forest School of Medicine, Winston-Salem, NC, USA; ^4^ Department of Social Sciences and Health Policy, Wake Forest School of Medicine, Winston-Salem, NC, USA; ^5^ Wake Forest School of Medicine, Winston-Salem, NC, USA; ^6^ Department of Radiation Oncology and Molecular Radiation Sciences, Johns Hopkins School of Medicine, Baltimore, MD, USA; ^7^ Division of Biostatistical Sciences, Wake Forest School of Medicine, Winston-Salem, NC, USA; ^8^ Department of Neurosurgery, Wake Forest School of Medicine, Winston-Salem, NC, USA; ^9^ Department of Cancer Biology, Wake Forest School of Medicine, Winston-Salem, NC, USA

**Keywords:** brain metastases, sociodemographics, stereotactic radiosurgery

## Abstract

**Background:**

Patient sociodemographic factors such income, race, health insurance coverage, and rural residence impact a variety of outcomes in patients with cancer. The role of brain metastasis at presentation and its subsequent outcomes have not been well characterized in this patient population.

**Results:**

Multivariate analysis revealed that median income lower than $50,000 was associated with higher presenting symptom grade for brain metastasis (mean RTOG grade 1.2 vs 1.0, SE = 0.1, *p* = 0.04) and higher chronic symptom grade (mean RTOG grade 1.3 vs 0.9, SE = 0.1, *p* = 0.002). Higher area-level median income was associated with a lower symptom grade at diagnosis of brain metastasis (*p* = 0.0008) and likelihood of hospitalization (*p* = 0.004). Other sociodemographic factors were not significantly associated with survival, neurologic death, or patterns of failure after stereotactic radiosurgery for brain metastases.

**Conclusions:**

Lower median income was associated with a greater symptom burden at the time of diagnosis and need for hospitalization for patients with brain metastases, suggesting a delayed time to presentation. These differences in symptom burden persisted during treatment.

**Methods:**

Between January 2000 and December 2013, we identified 737 patients treated with stereotactic radiosurgery for brain metastases. They were characterized by 4 sociodemographic factors: median income, race, rural-urban residence, and health insurance status. Clinical outcomes included stage at diagnosis, symptom grade at presentation, likelihood of hospitalization from brain metastasis, overall survival, local failure, distant brain failure, and neurologic death. Multivariate cox proportional hazards model for each outcome was performed controlling for age, sex, number of brain metastases, and dose to brain metastases.

## INTRODUCTION

Approximately 170,000 patients develop brain metastases from metastatic cancer in the United States each year [1]. The choice between stereotactic radiosurgery (SRS) and whole brain radiotherapy (WBRT) as the primary treatment option for brain metastases is controversial and with socioeconomic implications [2]. SRS is 3–4 fold more expensive than WBRT in the US [3]. However, the use of SRS without WBRT has led to improvements in cognitive outcomes and possibly even survival [4, 5]. SRS is generally reserved for patients with a limited burden of brain metastases [6] and thus patients whose disease is diagnosed earlier may be more likely to be eligible for and receive SRS. SRS is also not widely available in all clinical practices, especially in rural communities.

Low socioeconomic status and poor access to care (e.g. no health insurance) are associated with delays in diagnosis and inadequate care. For example, lower socioeconomic class has been associated with delayed presentation in both breast and rectal cancer [7]. Delays in care may lead to worsened survival [8, 9]. Innovations in medical treatment, such as SRS, may have the potential to exacerbate health disparities, as they may only be accessible to more advantaged groups and/or all groups may not benefit equally [10]. In the brain metastasis population, greater symptom burden presumably from delayed presentation has been shown to increase the likelihood of neurologic death [3].

Recent evidence suggests that there may be decreased utilization of SRS in patients of lower socioeconomic status [11]. There are no data, however, about potential disparities after SRS. Thus, we sought to examine clinical presentation and outcomes among patients with brain metastases by factors previously linked with cancer health disparities, including health insurance status, income, race and ethnicity, and rural residence. The goal of this single institution observational study was to examine the sociodemographic predictors of outcomes in patients with brain metastases who receive SRS. We hypothesized that among patients with brain metastases who undergo SRS, those with lower income would be more likely to have worse clinical outcomes.

## RESULTS

### Sample characteristics

Patient characteristics are shown in Table 1. The database compiled 737 patients treated from January 2000 to December 2013. There were 54% that were male and 89% were white. Medicare insurance participants made up 48% of the group. There were 37% who had private insurance, 12% had Medicaid, 2% were designated as having VA coverage, and the other 2% were either personal pay or no insurance. In regards to living areas, 71% of subjects lived in a metro area, 16% in a micropolitan designated area, and 13% were from rural areas.

**Table 1:** Baseline characteristics

Total	737 (100.0%)
Age [mean(sd)]	61.3 (12.8)
Gender	
Women	341 (46.3%)
Men	396 (53.7%)
Race	
Non-white	84 (11.4%)
White	653 (88.6%)
Primary Site	
Breast	102 (13.8%)
Colon	40 (5.4%)
Esophagus	17 (2.3%)
Lung	364 (49.4%)
Melanoma	117 (15.9%)
Other	29 (3.9%)
Renal/RCC	68 (9.2%)
Initial RTOG symptom grade	
0	195 (26.5%)
1	95 (12.9%)
2	223 (30.3%)
3	200 (27.1%)
4	24 (3.3%)
Insurance Status	
Medicaid	88 (11.9%)
Medicare	351 (47.6%)
None/Personal pay	14 (1.9%)
Private	269 (36.5%)
VA	15 (2.0%)
Number of Metastases [median(iqr)]	1.0 (1.0, 3.0)
Margin Dose [median(iqr)]	19.0 (17.0, 21.0)
Zip code Income Quartiles	
≤36,500	182 (24.7%)
36,500–44,000	189 (25.6%)
44,000–50,000	180 (24.4%)
>50,000	186 (25.2%)
RUCA	
Metro	527 (71.5%)
Micro	117 (15.9%)
Rural	93 (12.6%)

### Sociodemographic factors and stage at original diagnosis of cancer

Unadjusted ordinal logistic regression was performed to determine if sociodemographic factors were associated with stage at diagnosis (stage 4 or earlier stage) at time of first diagnosis of the metastatic cancer. White patients were more likely to be diagnosed with non-stage 4 disease at first diagnosis (OR 1.9, *p* = 0.02). No other sociodemographic factors were associated with stage at first diagnosis.

### Sociodemographic factors, symptom grade, and hospitalization due to brain metastasis

Multivariate analysis of factors associated with symptom grade at time of brain metastasis diagnosis revealed that estimated median income in the lower three quartiles was associated with a higher presenting symptom grade for brain metastasis (mean RTOG grade 1.2 vs 1.0, SE = 0.1, *p* = 0.04). No other sociodemographic factors including race, insurance status or rural status were significantly associated with symptom grade at time of diagnosis. Results of this analysis are summarized in Table 2.

**Table 2:** Multivariate analysis of predictors of RTOG symptom grade

	Estimated initial RTOG grade by income level		Estimated initial RTOG grade by income level	
	LSMean (SE)	*p*-value	LSMean (SE)	*p*-value
ZIP income Quartiles		0.0352		0.002
≤36,500	1.2 (0.1)		1.2 (0.1)	
36,500–44,000	1.1 (0.1)		1.2 (0.1)	
44,000–50,000	1.4 (0.1)		1.4 (0.1)	
>50,000	1.0 (0.1)		0.9 (0.1)	
RUCA		0.2339		0.2605
Metro	1.2 (0.05)		1.1 (0.06)	
Micro	1.3 (0.1)		1.4 (0.1)	
Rural	1.0 (0.1)		1.1 (0.1)	

Given the association between estimated income and brain metastasis symptom grade at diagnosis, a separate multivariate analysis of factors associated with chronic symptom grade revealed that estimated median income in the lower three quartiles was also associated with a higher symptom grade chronically (mean RTOG grade 1.3 vs 0.9, SE = 0.1, *p* = 0.002). No other sociodemographic factors including race, insurance status or rural status had a significant association with chronic symptom grade.

Given the association between income level and symptom grade, a multivariate logistic regression was performed to assess the effect of income on other clinically relevant factors including use of novel targeted systemic therapies, systemic disease status, presence of any neurologic symptoms, hospital stay and inpatient rehabilitation. This logistic regression revealed that mean estimated income was associated with the likelihood of hospitalization due to brain metastasis (*p* = 0.004). Results of this analysis are summarized in Table 3.

**Table 3:** Effect of estimated median income

Outcome *N* (%)	*N* = 732	Income Mean (sd)	*p*-value
Use of Targeted Agents^(a)^			0.9688
No	484 (66.1%)	45,610 (13,334)	
Yes	248 (33.9%)	45,615 (12,899)	
Systemic Disease^(a)^			0.0788
Stable	398 (54.4%)	44,948 (12,721)	
Progressive	260 (35.5%)	46,664 (13,766)	
Unknown^*^	74 (10.1%)	45,482 (13,438)	
Symptoms present at Dx			0.1926
No	196 (26.8%)	46,602 (13,490)	
Yes	536 (73.2%)	45,249 (13,058)	
Hospital Stay^(a)^			0.0040
No	368 (50.3%)	47,085 (13,703)	
Yes	364 (49.7%)	44,122 (12,470)	
Inpatient Rehab^(a)^			0.6404
No	702 (95.9%)	45,563 (13,064)	
Yes	30 (4.1%)	46,737 (15,845)	
Initial Symptom Grade^(b)^			0.0008
0	193 (26.4%)	46,690 (13,421)	
1	94 (12.8%)	48,967 (14,509)	
2	222 (30.3%)	45,798 (14,015)	
3	199 (27.2%)	43,548 (10,892)	
4	24 (3.3%)	39,187 (10,981)	
Time to Neurologic Death^(c)^			0.8833
Time to Local Failure^(c)^			0.8539
Time to Distant Failure^(c)^			0.8721

*p*-values in table are from (a) logistic regression, (b) linear model, or (c) Cox proportional hazards models. Income refers to the median income of the zip code of the participant.

### Effects of sociodemographic status on clinical outcomes

Multivariate analysis showed that none of the sociodemographic factors were associated with either time to death from any cause (overall survival) or time to neurologic death, including non-white race (*p* = 0.84, *p* = 0.67 respectively), lack of insurance (HR 1.51 with 95% CI 0.85 to 2.68, HR 1.11 with 95% CI 0.40 to 3.10), or rural-urban status. There was a significant association between survival and estimated local median income but without an increasing or decreasing trend across the four income quartiles.

Multivariate analysis showed that none of the assessed sociodemographic factors were associated with either local or distant brain failure, including non-white race (*p* = 0.67, *p* = 0.99 respectively), or lack of insurance (HR 1.04 with 95% CI 0.56 to 1.94, HR 1.51 with 95% CI 0.87 to 2.63). There was a significant association between failure and estimated local median income but without an increasing or decreasing trend across the four income quartiles.

## DISCUSSION

In this observational study of patients receiving SRS for brain metastases, we found that lower estimated income was significantly associated with more severe neurologic symptoms at presentation of brain metastasis, and increased likelihood of hospitalization due to brain metastasis, and that this greater symptom burden of lower income patients persisted after treatment. This suggests that barriers to care associated with income may lead to later clinical presentation of brain metastases and greater symptom burden. Time to clinical events (time to local/distant failure, neurological/overall death) in the current study was not significantly associated with race, lack of insurance, or rural-urban status. Although there was a significant association between survival and estimated local median income, there was no increasing or decreasing trend across the four income quartiles. Advanced metastatic disease typically has a grim overall prognosis with the risk of neurological death dependent on the number/location of both brain and systemic metastases [12]. Even if SRS is effective at treating brain metastases, such treatment may not be able to alter patient outcomes due to competing risk of death from other causes [13].

Our symptom burden results are similar to recent reports describing sociodemographic disparities in the setting of patients with brain metastases [11, 14]. Previous research has found that greater socioeconomic deprivation (including local median income) is associated with a delay in symptomatic presentation due to advanced cancer [7]. In that study from the United Kingdom, results of questionnaires suggested that in addition to geographic barriers to care, there was also a less awareness of the significance of symptoms by lower socioeconomic class [7]. This finding in addition to the findings of the current study would suggest that education of disparate populations with cancer of the symptoms of brain metastases may be advantageous.

In contrast to other studies, we did not observe associations between sociodemographic factors and patterns of failure after SRS. This difference may be due to patient selection bias based on institutional practice and referral patterns. In a multi-institutional analysis performed by several large volume cancer centers, Ayala-Peacock et al. reported that the non-white population had a significantly higher rate of developing new brain metastases after SRS [15]. This finding likely is a result of the inclusion of primary cancers with an enhanced propensity for brain metastases, such as triple negative breast cancer that are more prevalent in minority populations [16]. However, socioeconomic differences may also be causal. Also, novel systemic agents appear to improve control of extracranial disease and decreased distant brain failure [17]. However, these newer agents can be cost prohibitive to uninsured or underinsured patients.

There were several limitations to this study. This study was limited to those patients who received SRS; other patients who had delayed presentation and had neurological death prior to receiving SRS or were never referred to our tertiary care center are not in the sample. In addition, patients receiving SRS have already overcome barriers relating to health care access, potentially reducing variation by sociodemographic factors. We recognize that census data based methodology might not accurately represent the population context because of the decennial nature of census data, under count, population growth, and migration; however, this technique has been used with some degree of frequency in academic publications and has been shown in multiple studies to have a correlation to both true income and mortality [18]. Patients whose incomes were outliers for their ZIP code would dilute trends, though the large size of the cohort would likely negate most of that effect [19]. We also were unable to assess educational status which may be a potential confounding factor. There is a limitation to what factors can be abstracted out of an electronic medical record, limiting our ability to determine factors that might cause patients with lower income to have different outcomes. It is also possible that our study was under-powered to detect a difference in some of our clinical outcomes with less variation. In spite of the limitations, the results of this study suggest that income may be related to presentation for care for brain metastases and disparities in symptom burden may persist, despite receipt of best available treatments.

## CONCLUSIONS

Lower local median income was associated with greater symptom burden at the time of diagnosis and need for hospitalization among patients with brain metastases, suggesting a delayed time to presentation. These differences were maintained in the chronic symptom burden.

## METHODS

### Data acquisition

This study was approved by the Wake Forest Institutional Review Board. Data was reviewed and collected at the Wake Forest Comprehensive Cancer Center on 1052 patients treated with SRS between January 2000 to December 2013. Patients who received prior WBRT were excluded from the study because these patients were generally treated at a different point in their natural history than time of brain metastasis diagnosis. Patients with brain metastases from rare cancer types such as sarcoma, gynecologic cancers, and head and neck primary cancers were also excluded. Of the 1052 patients treated with SRS during this time period, 737 met study inclusion criteria. Patient clinical characteristics, sociodemographic factors and clinical outcomes were abstracted from the electronic health records (EHR) using structured abstraction forms and methods. Clinical factors collected to be correlated with sociodemographic factors included systemic disease status (progressive vs stable), presence of neurologic symptoms, use of targeted agents, in-patient hospitalization stay and in-patient rehabilitation. Symptoms at initial presentation and chronic symptoms were graded based on the RTOG Brain Grading scale.

### Sociodemographic characteristics

Race (white, non-white) and type of health insurance coverage (characterized as none, Medicare, private, Medicaid, VA/Tricare) were determined from the EHR. Due to small sample size, Hispanic ethnicity was not assessed as part of this study. While income could not be determined directly from the EHR, it was estimated based on the median income of the ZIP code in which the patient lived, as reported in the 2010 US Census. The sample was grouped into quartiles by local median income which corresponded to cut-off values at $36,500, $44,000, and $50,000. Residence was categorized by rural-urban commuting area (RUCA) codes using the ZIP code approximation [20]. These categories were defined as metropolitan areas within/adjacent to a built-up area with 50,000 or more population, micropolitan areas within/adjacent to a built-up area of 10,000 to 50,000 population, and rural areas with no associated built-up areas of more than 9,999 population.

### Outcomes assessment

Patient stage at diagnosis of cancer was determined from the EHR. Pre- and post-treatment symptom grade were determined based on the Radiation Oncology Group (RTOG) central nervous system (CNS) toxicity grading system.

After SRS, patients were followed with MRI approximately 2 months after initial SRS procedure, and then every 3 months subsequently as part of routine clinical care. Distant failures on follow-up MRI were defined as a new lesion that developed outside of the prior radiosurgical treatment volume. Local failures were defined as a pathologically-proven recurrence within the SRS treatment volume, or an increase in area of enhancement by 25% on an axial slice or serial increases in size of enhancement with corresponding increased perfusion on perfusion-weighted imaging.

### Statistics

Summary statistics were used to describe the sample and look at distributions of subjects within the socioeconomic groups. A Chi-Square was used to test for associations between local median income and rural-urban status. Bivariate linear (ANOVA) models were fit to test if socioeconomic factors had an association with severity of symptoms (RTOG grade). Multivariate Cox proportional hazard models were used to assess the influence of the socioeconomic factor after adjusting for age, sex, histology, number of brain metastases, and dose delivered to brain metastases for the outcomes of time to death (overall survival), time to neurologic death, time to local failure, and time to distant failure. All analyses were conducted using SAS 9.4 (SAS Institute, Cary NC).
